# Roasting‐Like Thermal Reconfiguration of Bioactive and Pigment Profiles in Pistachios From Distinct Geographical Origins

**DOI:** 10.1002/fsn3.72051

**Published:** 2026-06-25

**Authors:** G. Pedron, G. Barbieri, Y. Jaouhari, R. Perestrelo, J. S. Câmara, M. Bordiga

**Affiliations:** ^1^ Department of Pharmaceutical Science Università degli Studi del Piemonte Orientale “A. Avogadro” Novara Italy; ^2^ CQM—Centro de Química da Madeira Universidade da Madeira Funchal Portugal; ^3^ Departamento de Química, Faculdade de Ciências Exatas e Engenharia Universidade da Madeira Funchal Portugal

**Keywords:** antioxidant capacity, geographic origin, phenolic compounds, pigments, pistachios, roasting process

## Abstract

Roasting profoundly influences the phytochemical composition of pistachio ( *Pistacia vera* L.) kernels, yet the interaction between processing and geographical origin remains poorly understood. This study evaluated the impact of a dry roasting‐like treatment at 140°C for 45 min on the bioactive profile, pigments, and antioxidant capacity of pistachios from Iran, Spain, the USA, and Turkey. Raw and roasted samples subjected to a dry roasting‐like treatment were analyzed for total phenolic content (TPC), individual (poly)phenols by HPLC–DAD, antioxidant capacity using ABTS and DPPH assays, and pigments including chlorophylls and carotenoids. Results showed that geographical origin influenced all parameters. Among raw samples, Turkish pistachios exhibited the highest antioxidant capacity, with ABTS and DPPH values of 1.05 and 3.73 mg TE/g dw, respectively, whereas Iranian samples showed the highest TPC, at 2.5 mg GAE/g dw. The dry roasting‐like treatment induced marked compositional shifts, particularly in Turkish samples, with increases of 105% in ABTS, 255% in DPPH, and 255% in TPC. HPLC–DAD identified p‐hydroxybenzoic acid as the predominant phenolic compound. The treatment affected individual (poly)phenol concentrations differently depending on origin: total quantified levels increased in USA samples from 140 to 156 μg/g dw but decreased markedly in Turkish samples, from 665 to 241 μg/g dw. Specifically, gallic acid increased across all origins, likely due to the thermal hydrolysis of complex phenolic structures, whereas protocatechuic acid decreased. In terms of pigments, chlorophylls underwent extensive degradation, while carotenoids remained relatively thermally stable. Overall, the dry roasting‐like treatment triggered a compositional reconfiguration in which the degradation of thermolabile compounds may have been counterbalanced by the formation of Maillard reaction products, resulting in a net enhancement of functional quality. These findings elucidate the dynamic interaction between geographical origin and thermal processing and provide a scientific basis for promoting pistachios subjected to a dry roasting‐like treatment as a functional food contributing to the dietary intake of bioactive phytochemicals.

## Graphical abstract

Dry roasting‐like treatment (140°C for 45 min) induces a significant compositional reconfiguration of (poly)phenols, pigments, and antioxidant capacity in pistachio kernels, strictly dependent on their geographical origin (Iran, Spain, USA, and Turkey), ultimately enhancing their functional quality.

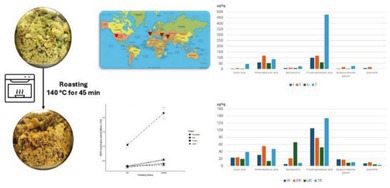

## 1 Introduction

As a key member of the Anacardiaceae family, the pistachio ( *Pistacia vera* L.) is not only a popular snack, consumed in various forms from raw to roasted and salted, but also serves as a crucial ingredient in numerous processed food applications. The economic importance of pistachios is reflected in the dramatic increase in global production, which more than doubled from approximately 640,000 tons in 2010 to 1.3 million tons by 2023. In 2023, the U.S.A. produced approximately 700,000 tons, followed by Iran and Turkey. This growth has fuelled a highly competitive market, characterized by shifts in global leadership. This dynamic demonstrates the volatility and high value of the global pistachio market (Mandalari et al. 2022; Pedron et al. 2025; FAOSTAT 2025).

Beyond their taste, pistachios offer notable nutritional and functional benefits. They are characterized by a low level of saturated fatty acids and sugars, while being a rich source of unsaturated fatty acids and dietary fiber. In addition, pistachios represent a rich source of bioactive compounds, including tocopherols, carotenoids, and (poly)phenols, which collectively contribute to their antioxidant properties and associated health‐promoting effects (Dreher 2012).

(Poly)phenols are a broad class of secondary plant metabolites renowned for their diverse bioactive properties, most notably their potent antioxidant capacity. These compounds are chemically defined as organic molecules characterized by the presence of one or more aromatic rings with attached hydroxyl groups. They are extensively categorized into several major subclasses, including phenolic acids (e.g., hydroxybenzoic acids, hydroxycinnamic acids), flavonoids (e.g., flavones, flavanols, isoflavones, flavanones, anthocyanins), stilbenes (e.g., resveratrol, piceatannol), and lignans (e.g., sesame, pinoresinol, sinol, enterodiol) (Handique and Baruah 2002; Zagoskina et al. 2023).

Numerous epidemiological studies have demonstrated that (poly)phenolic molecules play a crucial role in protecting cells and biological metabolites against oxidative stress (Dini and Grumetto 2022). It is well established that (poly)phenols can scavenge radical species or prevent their formation by inhibiting the activity of pro‐oxidative enzymes and chelating transition metals that act as catalysts for oxidation. Their capacity to neutralize free radicals is primarily attributed to the presence of hydroxyl groups on the aromatic ring system, which allow for the donation of a hydrogen atom or an electron to the radical, subsequently stabilizing it through resonance delocalization. This mechanism effectively interrupts the oxidative chain reaction (Gulcin 2020; Leyane et al. 2022). Within food systems, (poly)phenols are essential for inhibiting lipid peroxidation and amino acid oxidation, thereby preserving the original flavor, color, texture, and health benefits of the product (Gulcin 2020).

Pistachios, particularly in their raw form, are rich in phenolic compounds, but their distribution and concentration can vary significantly with geographical origin and on the subsequent technological processes. The pistachio seed's phenolic profile is dominated by quercetin‐3‐O‐rutinoside and genistein, while compounds like gallic acid and catechin are present in lower quantities. In stark contrast, the pistachio skin is an exceptionally rich source of these compounds. It contains a very high concentration of cyanidin‐3‐O‐galactoside, alongside significantly higher levels of gallic acid and catechin compared to the seed (Tomaino et al. 2010).

The roasting process is crucial for commercial nut quality, defining their final sensory profile, texture, and stability through thermally induced reactions, predominantly the Maillard reaction. Distinct roasting technologies (e.g., conventional ovens, infrared, and microwave) impart unique characteristics, the precise selection and control of the roasting method are fundamental, as they dictate the nut's thermal history and consequently, its final physicochemical properties and overall product consistency. Furthermore, roasting significantly changes the pistachio's polyphenolic composition and antioxidant properties, outcomes depend on the final composition being a complex interplay of the pistachio variety, geographical origin, and specific roasting parameters. For instance, a study conducted by Rodríguez‐Bencomo et al. (2015) demonstrated that roasting pistachio kernels at 160°C led to a notable increase in the concentration of phenolic compounds, namely protocatechuic acid, catechin and chlorogenic acid, particularly after a longer roast (40 min). This observed increase in detectable polyphenols is likely due to the thermal breakdown of the pistachio's plant cell structures, which releases bound phenolic compounds, making them more accessible for extraction and detection. Consequently, roasting serves not only as a crucial flavor and texture development step but also as a potentially useful method to enhance the concentration and potential bioavailability of certain beneficial compounds in pistachios (Bhinder et al. 2019; Chandrasekara and Shahidi 2011; Pedron et al. 2025; Rabadán et al. 2018; Rodríguez‐Bencomo et al. 2015; Şen and Gökmen 2022; Suri et al. 2019; Zhang et al. 2006).

The visual quality of pistachio kernels, particularly their color, is a paramount factor governing both consumer acceptance and market valuation. Pistachio kernels typically exhibit a spectrum of hues ranging from vibrant green to yellow, a characteristic influenced by a complex interplay of factors, including cultivar, geographical origin, maturity stage, and subsequent post‐harvest processing techniques. This characteristic color profile is primarily derived from two major classes of liposoluble pigments, largely concentrated within the outer seed coat: chlorophylls (a and b), which are the dominant contributors to the desirable green tones (150 μg/g in raw kernels), and carotenoids, such as lutein (18 to 52 μg/g) and β‐carotene (1.8 μg/g), which are responsible for the yellow to orange hues. However, thermal processing, an essential step in preparing pistachios for consumption, critically impacts this pigment profile. During heating, chlorophylls are highly susceptible to degradation, undergoing pheophytinization, the displacement of the central magnesium atom to form brownish‐green pheophytins and subsequent pyro‐derivatives, which collectively lead to a significant loss of green color intensity. In contrast, carotenoids, particularly lutein, exhibit comparatively greater thermal stability, allowing the yellow background hues to persist even as the more labile green tones diminish (Bellomo and Fallico 2007; D'Evoli et al. 2015; Dini et al. 2019; Pumilia et al. 2014; Rabadán et al. 2018; Shang et al. 2025).

A review of current literature reveals that most studies on pistachio thermal processing utilize whole kernels to simulate traditional commercial roasting (Hojjati et al. 2015; Kahyaoglu 2008; Ling et al. 2016; Rodríguez‐Bencomo et al. 2015; Yuan et al. 2022). While these studies are industrially relevant, the uneven heat distribution within a whole kernel can mask the specific chemical kinetics of (poly)phenol transformation. In the present study, we deliberately opted to roast the pistachios in powdered form. This approach was chosen to maximize the surface area exposed to thermal treatment, thereby facilitating the exhaustive liberation of bound phenolic compounds from the cell wall matrix (e.g., complexed with fibers or polysaccharides), as suggested by recent evidence (Akele et al. 2024; Antony and Farid 2022; Jiang et al. 2023; Mantia et al. 2023). By utilizing a powdered matrix, we aimed to investigate the “upper limit” of antioxidant potential release and structural degradation under controlled laboratory conditions across various geographical origins. This methodology provides a unique perspective on the fundamental chemical stability and reactivity of pistachio (poly)phenols that may be partially shielded in whole‐kernel roasting.

This investigation will provide a comprehensive understanding of how powder roasting under controlled oven conditions and geographical origin jointly influence the nutritional and phytochemical composition of pistachios.

## 2 Methods and Materials

### 2.1 Solvents and Reagents

All solvents used for High‐Performance Liquid Chromatography (HPLC), namely methanol and acetonitrile, were of HPLC grade. Formic acid (50%, LC–MS grade) was also employed.

All chemicals used were of analytical grade. Reagents: free radical scavenging agents, including 2,2‐diphenyl‐1‐picrylhydrazyl (DPPH) and 2,2′‐azino‐bis(3‐ethylbenzothiazoline‐6‐sulfonic acid) (ABTS), along with potassium hydroxide (KOH), were purchased from Sigma‐Aldrich (Merck KGaA, Darmstadt, Germany).

Standards: The following polyphenol reference standards were obtained from Sigma‐Aldrich (Merck KGaA, Darmstadt, Germany): gallic acid, protocatechuic acid, p‐hydroxybenzoic acid, quercetin, epicatechin, epigallocatechin, and epigallocatechin gallate.

Solvents: HPLC‐grade petroleum ether (PE), tetrahydrofuran (THF), and dichloromethane (DCM) were sourced from Sigma‐Aldrich (Merck KGaA, Darmstadt, Germany).

Ultrapure water (resistivity 18.2 MΩ·cm at 25°C) used in the preparation of all solutions was produced using an ELGA PURELAB Ultra system (M‐Medical, Milan, Italy).

### 2.2 Samples Preparation and Processing of Pistachios

In this study, the composition of raw and dry roasting‐like treatment pistachios from four distinct geographical origins: Iran (I), Europe from Spain (E), the United States from California (U), and Turkey (T) was analyzed.

For each origin, 500 g of each raw pistachio samples (2023 harvest) were supplied by UNIGRÀ S.p.A. (Conselice, Ravenna, Italy) and collected in 2024. While these samples represent major global production regions, specific details regarding cultivar, pedoclimatic conditions, and precise post‐harvest handling were unavailable.

To ensure maximum data homogeneity, a standardized sampling and extraction protocol was adopted. For each geographical origin (USA, Europe, Turkey, and Iran), a representative sample of 50 g of pistachios was randomly collected from the initial batch and ground using a laboratory mixer mill (MM40, Retsch GmbH, Haan, Germany) to obtain a homogeneous powder. Subsequently, for each origin, a 20 g aliquot of the homogenized powder was subjected to thermal treatment at 140°C for 45 min in a ventilated oven (FKV Srl, Torre Boldone, Bergamo, Italy), following the protocol reported by (Kahyaoglu 2008). While the roasting process was carried out as a single event for each batch, the subsequent chemical extraction procedure for both raw and roasted samples was performed in triplicate (*n* = 3) to serve as technical replicates. Spectrophotometric assays (DPPH, ABTS, and pigments) were performed in triplicate over three consecutive days to evaluate inter‐day technical reproducibility, and final results were expressed as means ± standard deviation. Conversely, polyphenol characterization via HPLC was performed once for each independent extraction replicate.

Accordingly, the codes I, E, U, and T refer to the raw samples from Iran, Europe, the USA, and Turkey, respectively, while IR, ER, UR, and TR denote their dry roasting‐like treatment counterparts.

### 2.3 (Poly)phenols Extraction

Firstly, the lipids fraction of raw and roasted pistachios was removed through a semi‐automatic Soxhlet apparatus (BÜCHI Extraction System B‐811) using dichloromethane as the extractant solvent for 6 h. Dichloromethane was selected as the extraction solvent due to its high efficiency in exhausting the complex lipid matrix and its low boiling point (approximately 40°C), which ensures the preservation of thermolabile phenolic compounds during the 6 h process. After that, the non‐lipids fraction was left under the extractor hood overnight to ensure complete solvent evaporation.

Subsequently, (poly)phenols were extracted from the defatted fraction following the procedure of (Bordiga et al. 2015; Goli et al. 2005), with some little adjustment. Briefly, 1 g of finely ground sample was mixed with 30 mL of methanol, vortexed, and sonicated in an ultrasonic bath (Branson 1510) for 5 min, followed by agitation on an orbital shaker for 1 h. The resulting suspension was then subjected to centrifugation at 10,000 rpm for 10 min at 10°C, and the supernatant was collected. The extraction was repeated with 15 mL of methanol and shaking for 30 min, after which both supernatants were combined in an amber flask. The solvent was evaporated under reduced pressure through rotary evaporator (Rotavapor R‐210); the bath temperature was set at 35°C. To ensure the total removal of solvent residues, the vacuum was maintained at < 15 mbar for an additional 15 min after the visible solvent had evaporated, ensuring the residue reached a constant weight and a solid consistency. The residue was reconstituted in 1.0 mL of methanol, scraped, filtered through a 0.45 μm RC filter, and transferred to a flask of 5 mL. The flask was rinsed with 1.5 mL of methanol, and the wash was combined with the initial extract for subsequent analysis.

### 2.4 Total Phenolic Content (TPC)

The TPC of the pistachio extracts was determined using the Folin–Ciocalteu assay, following the procedure described by Bordiga et al. (2015), Goli et al. (2005), with minor modifications. The TPC was calculated by interpolating the sample absorbance data onto the linear gallic acid calibration curve (*R* ^2^ = 0.998) read at 760 nm in a UV–Vis spectrophotometer (Shimadzu UV‐1900 model, Shimadzu, Beijing, China). Results were expressed as milligrams of gallic acid equivalents (GAE) per gram of sample on a dry weight (dw), mg GAE/g dw. All determinations were performed in triplicate.

### 2.5 (Poly)phenol Characterization Through HPLC‐DAD

Chromatographic analysis was performed using a Shimadzu LC‐20A Prominence chromatographic system equipped with a diode array detector (DAD; SPD‐M20A). Separation was carried out on a Luna C18 column (150 × 2.0 mm, 5 μm particle size; Phenomenex, Torrance, CA, USA), as reported by Jaouhari, Ferreira‐Santos, et al. (2024). The mobile phase consisted of ultrapure water containing 0.1% (v/v) formic acid as eluent A and acetonitrile containing 0.1% (v/v) formic acid as eluent B. Elution was performed at a constant flow rate of 0.4 mL/min. The gradient program was as follows: 8%–18% B over 40 min, 18%–40% B over 12 min, 40%–75% B over 3 min, isocratic elution at 75% B for 10 min, 75%–8% B over 3 min, followed by re‐equilibration at 8% B for 12 min. The total run time was 80 min. Chromatograms were recorded at 280 and 330 nm.

The resulting linear regression equations and coefficients of determination (*R* ^2^) are summarized below:

$$\text{Gallic acid}:y=39731x-11015;R^{2}=0.998$$

$$\text{Protocatechuic acid}:y=21368x-6072.8;R^{2}=0.999$$

$$\text{Epicatechin}:y=9439x-0.6607;R^{2}=0.999$$

$$p-\text{Hydroxybenzoic acid}:y=16204x-736.65;R^{2}=0.999$$

$$\text{Epigallocatechin gallate}:y=3440.8x-5227.5;R^{2}=0.999$$

$$\text{Quercetin}:y=4799.7x-7840.4;R^{2}=0.998$$

### 2.6 Antioxidant Capacity Assay

The antioxidant capacity of the pistachio extracts was determined in vitro using DPPH^•^ and ABTS^•+^ radical scavenging assays, following the methodologies described by (Jaouhari, Disca, et al. 2024; Locatelli et al. 2009) with slight modifications. To quantify the antioxidant activity, a Trolox calibration curve was generated. A stock solution was prepared by dissolving 1 mg of Trolox in 1 mL of methanol (1 mg/mL). A seven‐point dilution series was then prepared by transferring 1, 2, 4, 6, 8, 10 and 12 μL of the stock solution into separate tubes and adjusting the final volume to 1 mL with methanol. For the DPPH assay, an aliquot of 700 μL from each standard or sample extract was mixed with 700 μL of a methanolic DPPH• solution. All calibration points and samples were analyzed in triplicate to ensure analytical reproducibility. Following a 30 min incubation period in the dark at room temperature, the absorbance was measured at 515 nm (for DPPH) and 734 nm (for ABTS) using a spectrophotometer. The results were expressed as milligrams of Trolox Equivalents per 100 g of dry weight (mg TE/100 g DW).

The resulting linear regression equation and coefficients of determination (*R* ^2^) are summarized below:

$$y=12.993x+2.9461;R^{2}=0.992$$

### 2.7 Chlorophyll and Carotenoids Content

The total content of chlorophyll (a + b) and total carotenoids was determined by spectrophotometric assay, following the procedure established by Dini et al. (2019) with minor modifications. The extraction and quantification procedure were performed on three different days to account for analytical and temporal variation. Each daily measurement was performed in triplicate, resulting in a total of nine analytical determinations per sample. The final pigment contents (total chlorophylls a + b and total carotenoids) were expressed as mg/kg of pistachio (dry weight basis).

### 2.8 Statistical Analysis

Statistical analyses were performed using R software (version 4.4.3). All experimental data are expressed as mean ± standard deviation (*n* ≥ 3).

Prior to performing the Two‐Way ANOVA, the homogeneity of variance was evaluated for all parameters using Levene's test. The assumption of homoscedasticity was verified for the majority of the analyzed variables (*p* > 0.05). For the spectrophotometric antioxidant assays (DPPH and ABTS), where a significant Levene's test was observed (*p* < 0.05), the robust nature of the Two‐Way ANOVA was relied upon, which is statistically guaranteed by the perfectly balanced experimental design (*n* = 3 for each experimental group).

To evaluate the influence of the independent variables, a Two‐Way Analysis of Variance (ANOVA) was applied, considering geographical origin and thermal treatment status (raw vs. dry roasting‐like treatment) as fixed factors, including their interaction term. When the ANOVA indicated significant effects (*p* < 0.05), Tukey's Honestly Significant Difference (HSD) post hoc test was employed for multiple comparisons, allowing for a comprehensive evaluation across all sample groups.

Furthermore, One‐Way ANOVA was utilized to specifically assess differences among samples within the same processing state (raw or dry roasting‐like treatment) from different origins.

To visualize the relationship between variables and the potential synergy between factors, interaction plots were generated using the ggplot2 package. This integrated approach was selected to provide both a rigorous statistical confirmation of the differences and a clear graphical interpretation of how thermal treatment differentially impacts the chemical profile of pistachios based on their provenance.

Finally, multivariate analysis was executed, specifically leveraging the FactoMineR and factoextra packages for the computation and graphical rendering of Principal Component Analysis (PCA) to further discriminate samples based on their origin and processing state.

## 3 Results and Discussion

### 3.1 Phenolic Content

**FIGURE 1:**
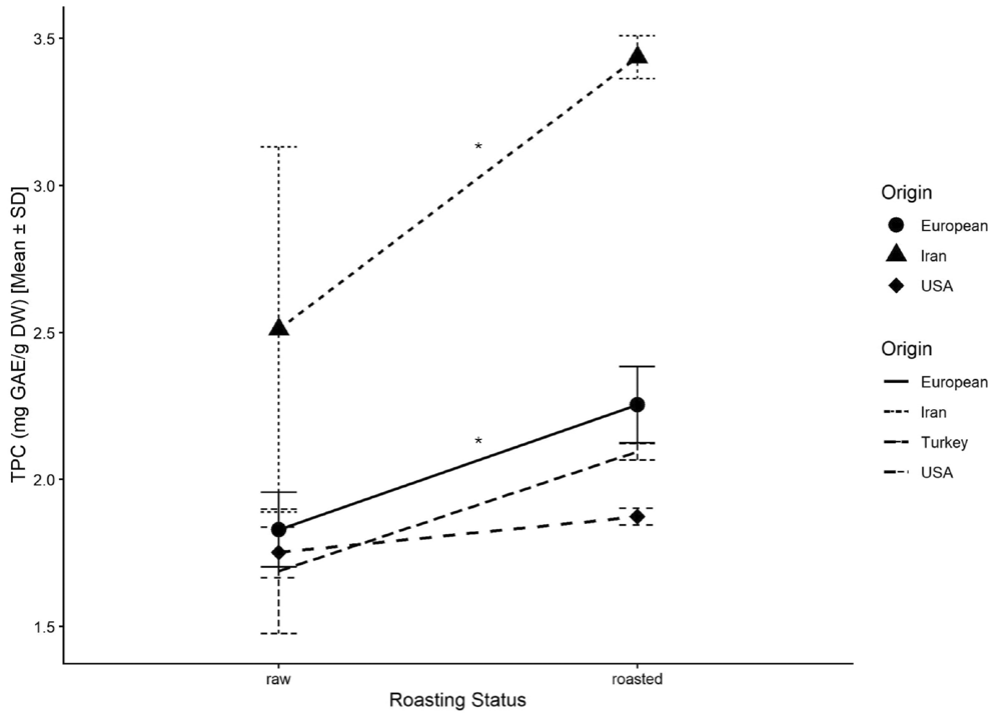
Interaction effect of geographic origin and roasting status on TPC (mg GAE/g dw). Data are presented as mean ± standard deviation (*n* = 3). Asterisk indicate significant differences between raw and roasted status within the same geographic origin (*p < 0.01), as determined by Two‐way analysis of variance (ANOVA) followed by Tukey's post hoc test.

The total phenolic content (TPC) of the samples varied notably according to geographical origin and processing conditions, as shown in Table 1 and Figure 1. Among the raw samples, the highest TPC was observed in the Iranian sample (I, 2.5 ± 0.6 mg GAE/g dw), followed by those from Europe (E, 1.8 ± 0.1), USA (U, 1.75 ± 0.09), and Turkey (T, 1.7 ± 0.2). No significant difference (*p* > 0.05) in TPC content was observed between Iranian and European samples, as well as among European, US, and Turkish samples.

**TABLE 1:** Data of TPC (mg GAE/g dw) and the relative change (Δ% TPC).

Sample	Raw (mg GAE/g dw)	Dry roasting‐like treatment (mg GAE/g dw)	Δ% TPC
I	2.50 ± 0.60^a^	3.44 ± 0.07^a^	+115
E	1.80 ± 0.10^a^	2.30 ± 0.10^b^	+27.9
U	1.75 ± 0.09^a^	1.87 ± 0.03^bc^	+95.2
T	1.70 ± 0.20^a^	2.09 ± 0.03^c^	+255

*Note:* Values are expressed as mean ± standard deviation (*n* = 3). Different superscript letters within the same column indicate statistically significant differences (*p* < 0.05). Origin codes: I, Iran; E, Spain; U, USA; T, Turkey.

The geographical variation observed in the TPC in this study is consistent with findings reported, which have documented between 1.3 and 3.5 mg GAE/g dw (Moreno‐Rojas et al. 2022). In contrast, the present data are lower than those reported by Kornsteiner et al. (2006), who detected TPC content between 4.9 and 14.4 mg GAE/g dw, with a mean value of 8 mg GAE/g dw. Such variability has been consistently linked to differences as well as to environmental and agronomic factors influencing phenolic biosynthesis. Furthermore, harvest conditions, such as maturity stage, drying processes, and storage, are also reported to significantly influence the final phenolic concentration and antioxidant activity of samples (Bolling et al. 2010; Kornsteiner et al. 2006; Moreno‐Rojas et al. 2022; Tsantili et al. 2011).

The roasting process consistently induced the most significant changes across all measured antioxidant parameters. Specifically, roasting markedly enhanced the TPC content of all pistachio samples. The most pronounced increase was observed in the Iranian sample, where the TPC rose from 2.51 to 3.44 mg GAE/g dw, representing an impressive 36.9% increase. This was followed by the European and Turkish samples, which exhibited increases of 23.2% and 14.1%, respectively. In stark contrast, the USA pistachios showed only a slight improvement, with a 6.99% increase in TPC. No significant difference (*p* > 0.05) in TPC content was observed among Europe, USA, and Turkish dry roasting‐like treatment samples.

The observed increase in TPC levels in our samples following heat treatment aligns with several studies in the literature concerning nuts and related matrices. Bagheri et al. (2016) reported a significant increase in TPC, in peanuts after roasting, with values rising from 0.76 mg GAE/g in the raw samples to 1.08 mg GAE/g in the roasted ones. Similarly, Hojjati et al. (2015) demonstrated that the processing method markedly effect the final antioxidant capacity of pistachios. Their study revealed no significant change in TPC when nuts underwent conventional hot‐air roasting compared to the raw sample (16.2 mg GAE/g). However, when the pistachios were roasted using microwave energy (640 W for 4 min), the TPC showed a notable increase to 20.7 mg GAE/g. In addition, Mustafa et al. (2021) observed that roasting enhanced the TPC of chestnuts from 8.98 mg GAE/G in raw samples to 9.43 mg GAE/g after roasting.

The observed increase in TPC following the roasting process can be attributed to a complex balance between the thermal degradation of specific thermolabile phenolics and the simultaneous liberation of bound compounds. Thermal treatment facilitates the breakdown of complex polyphenolic structures and the cleavage of bonds between phenolic acids and the cell wall matrix (e.g., lignin or polysaccharides), leading to an increase in free, extractable (poly)phenolic compounds. Furthermore, the Maillard reaction, occurring between free amino acids and reducing sugars at elevated temperatures, contributes significantly to this trend. This reaction leads to the synthesis of melanoidins and other neo‐formed compounds, which are well documented to possess strong reducing power and antioxidant activity, thereby inflating the total (poly)phenolic values measured via the Folin–Ciocalteu assay (Akele et al. 2024; Antony and Farid 2022).

Conversely, our results contradict the findings of Yuan et al. (2022), who reported a decrease in TPC following roasting, from 4.79 mg GAE/g in the raw sample to 4.47 mg GAE/g in pistachios roasted sample.

**FIGURE 2:**
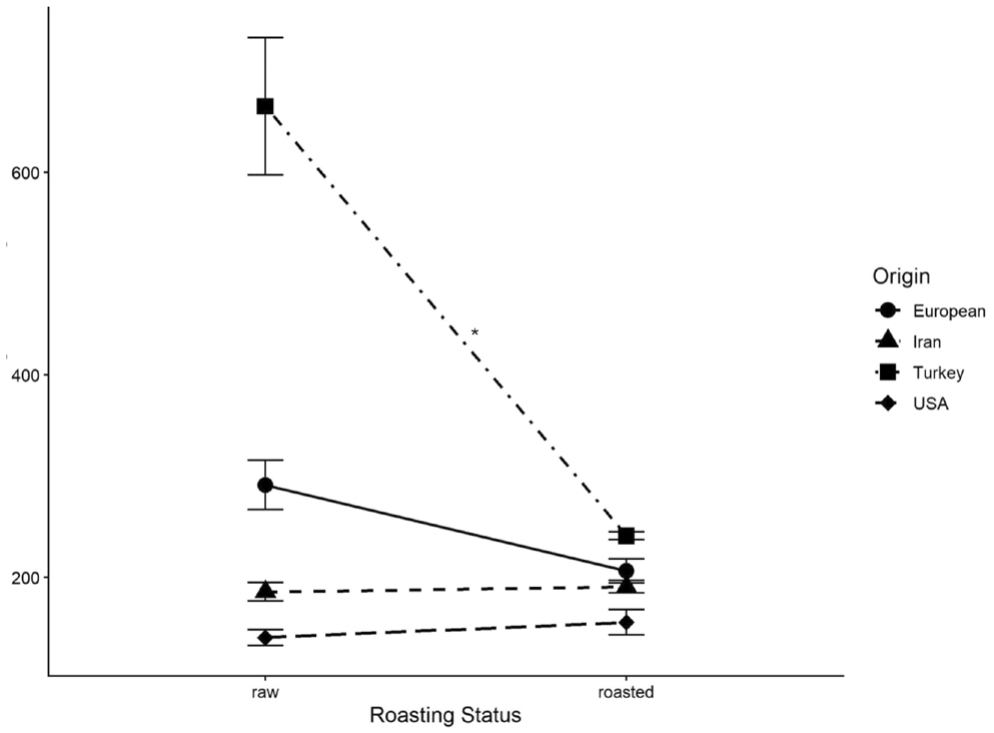
Interaction effect of geographic origin and roasting status on ∑ (poly)phenol content μg/g dw. Data are presented as mean standard deviation (*n* = 3). Asterisks indicate significant differences between raw and roasted status within the same geographic origin (*p < 0.01), as determined by Two‐way analysis of variance (ANOVA) followed by Tukey's post hoc test.

The qualitative and quantitative (poly)phenolic profiles, determined by HPLC–DAD, are summarized in Table 2. Figure 2 illustrates the interaction plot between geographic origin and roasting status on the total (poly)phenol content.

**TABLE 2:** Concentration of main (poly)phenol quantified through HPLC‐DAD (μg/g dw).

Sample	Gallic acid		Protocatechuic acid		Epicatechin		*p*‐Hydroxybenzoic acid		Epigallocatechin gallate		Quercetin		∑ (poly) phenol	
I	5.46 ± 0.4^b^		59,8 ± 1^c^		9.16 ± 0.5^b^		98.1 ± 6^b^		6.82 ± 0.7^c^		7.38 ± 0.8^b^		185 ± 9.1^bc^	
E	10.1 ± 0.8^b^		118 ± 11^a^		11.1 ± 0.2^b^		116 ± 10^b^		17.5 ± 0.3^b^		18.4 ± 1.3^a^		291 ± 24^b^	
U	6.93 ± 0.2^b^		51.7 ± 0.6^c^		7.47 ± 0.1^b^		59.1 ± 4^b^		5.97 ± 0.5^c^		3.66 ± 0.2^b^		140 ± 7.8^c^	
T	44.6 ± 4^a^		89.5 ± 5^b^		25.5 ± 2^a^		477 ± 57^a^		26.1 ± 0.8^a^		4.98 ± 0.4^b^		665 ± 67^a^	

	Gallic acid	Δ%	Protocatechuic acid	Δ%	Epicatechin	Δ%	*p*‐Hydroxybenzoic acid	Δ%	*p*‐Hydroxybenzoic acid	Δ%	Quercetin	Δ%	∑ (poly) phenol	Δ%
IR	23.5 ± 0.5^b^	337	30.5 ± 0.7^dc^	−48.1	4.85 ± 0.5^c^	−46.9	106 ± 4^b^	7.99	18.8 ± 1^a^	175	7.08 ± 0.2^b^	−4.04	191 ± 6.1^bc^	2.71
ER	23.7 ± 2^b^	134	55.4 ± 3^a^	−53.1	20.7 ± 2^b^	86.7	78.7 ± 3^c^	−32.1	17.5 ± 1^a^	−0.11	10.1 ± 0.4^a^	−45.4	206 ± 12^ab^	−29.2
UR	19.6 ± 0.4^b^	183	13.7 ± 1^d^	−73.3	66.2 ± 1^a^	786	52.4 ± 3^d^	−11.3	8.59 ± 0.3^b^	43.7	4.27 ± 0.3^c^	16.7	155 ± 12^c^	22.3
TR	39,4 ± 2^a^	−11.8	47.1 ± 0.8^b^	−46.8	8.24 ± 0.3^c^	−66.3	134 ± 2^a^	−71.90	9.89 ± 0.9^b^	−62.0	2.14 ± 0.03^c^	−46.4	241 ± 3.8^a^	−63.7

*Note:* Data are presented as mean ± standard deviation (*n* = 3). Statistical significance is indicated by different letters for each column, where values sharing the same letter are not significantly different (*p* < 0.05). Origin codes: I, Iran; E, Spain; U, USA; T, Turkey. IR, dry roasting‐like treatment Iran; ER, dry roasting‐like treatment Spain; UR, dry roasting‐like treatment USA; TR, dry roasting‐like treatment Turkey.

The data clearly indicate that geographical origin exerts a significant influence on both the composition and total concentration of (poly)phenolic compounds in raw pistachios. Among the evaluated samples, the Turkish raw pistachios exhibited the highest total (poly)phenol content (665 μg/g dw), followed by those from European (291 μg/g dw), the Iranian (185 μg/g dw), and USA (140 μg/g dw).

Following the roasting process, a marked decrease in total (poly)phenol concentration was observed in Turkish (−63.8%) and in European raw pistachios (−29.2%). In contrast, a slight increase USA (+10.8%) and in Iranian samples (+2.7%) after roasting process.

The observed variability in both concentration and qualitative profile across different geographical origins, consistent with findings reported for other nut species, can be attributed to intrinsic factors such as cultivar genetics and extrinsic factors including soil characteristics, climatic conditions, and harvest practices (Król et al. 2020; Locatelli et al. 2015; Mandalari et al. 2022; Pedron et al. 2025; Tsantili et al. 2010).

p‐Hydroxybenzoic was identified as the predominant (poly)phenolic compound in the raw pistachio samples, followed by acid and protocatechuic acid. Gallic acid, epicatechin, epigallocatechin gallate, and quercetin were also detected at lower concentrations.

Regarding p‐hydroxybenzoic acid, exhibiting the highest concentration in Turkish pistachios (477 μg/g dw), followed by the European (116 μg/g dw), Iranian (98.1 μg/g dw), and USA (59.1 μg/g dw) samples. The concentrations reported herein are considerably higher than those described by Moreno‐Rojas et al. (2022), who detected less than 4.06 μg/g across eleven Andalusia varieties, and La Camera et al. (2018), who reported 1.7 μg/g in Californian pistachios. Conversely, the high concentrations of protocatechuic acid in the European (118 μg/g dw) and Turkish (88.5 μg/g dw) samples are consistent with the values reported by Liu et al. (2014). Although the Iranian (58.8 μg/g dw) and USA (51.7 μg/g dw) samples exhibited comparatively lower concentrations, these are still substantially higher than the 10.1 μg/g previously reported for USA pistachios by La Camera et al. (2018). Moreover, Rodríguez‐Bencomo et al. (2015), reported a lower concentration of 31.4 μg/g in Turkish pistachios sample, reinforcing the substantial geographical variability observed in phenolic acid composition. Nevertheless, a recent study by Ripari Garrido et al. (2024) confirm notable variations in the concentration of key phenolic compounds among pistachios from different countries.

For instance, gallic acid concentration varied widely, with USA grown pistachios exhibiting the highest concentration (209.3 μg/g dw), followed by Argentina (83 μg/g dw), and Italy (14.1 μg/g dw). A similar trend was observed for epicatechin, which was most abundant in USA samples (229.1 μg^/^g), while Italian and Argentinian pistachios contained 104.8 and 27.5 μg/g, respectively. The most significant difference was observed in catechin concentration: USA pistachios contained a remarkably high concentration of 1795.7 μg^/^g, far exceeding those detected in Italian (379.9 μg/g) and Argentinian (156 μg/g) samples. Interestingly, genistein was exclusively detected in Italian pistachios (69.9 μg/g) and was absent from both USA and Argentinian samples (Ripari Garrido et al. 2024).

For the total flavonoid content (sum of quercetin, epicatechin and epigallocatechin gallate), Turkish pistachios sample exhibited the highest concentration (54.5 μg/g dw), followed by the European (47.1 μg/g dw), Iran (23.4 μg/g dw), and USA (17.1 μg/g dw). In raw European pistachios, the concentration of epigallocatechin (17.5 μg/g dw) and quercetin (18.4 μg/g dw) was particularly noteworthy. The quercetin levels observed in this study are comparable to those reported by Younis et al. (2024) for Turkish samples (1.5 μg/g dw), although lower than those found in the Iranian and European pistachios analyzed in the current study.

The roasting process induced compound‐specific changes. Most strikingly, regarding p‐hydroxybenzoic acid, an increase was observed only in Iranian pistachios (+176%), while all other samples experienced a decrease (Turkey: −71.9%, European: −32.2%, USA: −11.3%). Protocatechuic acid decreased constantly across all samples, with the largest reduction in the USA sample (−73.4%). This consistent decrease contrasts with the increase reported by Rodríguez‐Bencomo et al. (2015) and Yuan et al. (2022), a discrepancy potentially due to differences in roasting conditions (whole vs. ground nuts).

Among minor poly(phenols), the roasting process induced variable but significant changes, highlighting contrasting responses based on geographical origin. Gallic acid concentration increased markedly in all samples except Turkey, with the most substantial rise observed in the Iranian sample (+337%), a finding consistent with Mandalari et al. (2013). Epicatechin showed the most pronounced increase in the USA sample (+786%) and the European sample (+86%), while it decreased significantly in Turkey (−66.3%) and Iran (−47%); this general decreasing trend aligns with the observation of Mandalari et al. (2013). Epigallocatechin gallate concentration increased in the Iranian (+175%) and USA (+43.7%) samples, but showed a severe reduction in the Turkish sample (−62%), consistent with the findings of Yuan et al. (2022).

The data, in general, demonstrate a consistent decreasing trend in the total poly(phenol) content following the roasting process across European (−60.4%), USA (−51.2%), and Turkish (−29.0%) pistachios. This pattern aligns with several reports describing the impact of heat treatment on other nut matrices, including hazelnuts, peanuts, and cashews, which similarly exhibit a net reduction in total phenolic content. These declines are primarily attributed to the thermal degradation and oxidation of labile phenolic compounds, particularly those concentrated in the thin skin of the nut, which are highly susceptible to heat damage. However, the slight overall increase observed in the Iranian sample (+10.4%) and the specific substantial increases in individual compounds (such as gallic acid and Epigallocatechin gallate in Iranian and USA samples) highlight a distinct and compound‐specific response to roasting.

The observed increase in specific bioactive compounds following the roasting process is likely attributable to the thermal hydrolysis and subsequent breakdown of complex, insoluble phenolic matrices, such as tannins and glycosides. This degradation facilitates the liberation of bound phenolics, transitioning them into an extractable and quantifiable state. Antony and Farid (2022) suggest that thermal energy induces the cleavage of ether and ester bonds between lignin and phenolic acids, as well as the partial depolymerization of the lignin fraction itself. These authors noted that phenolic acid concentrations post‐thermal treatment could reach levels twice as high as those of the free phenolic fraction in the raw matrix.

Furthermore, temperature‐dependent shifts in specific (poly)phenol have been observed in similar matrices. For instance, Mantia et al. (2023) reported that epicatechin levels in chocolate increase during low‐to‐medium roasting (80°C–100°C), whereas at higher temperatures (130°C), the total antioxidant capacity is maintained by a synergistic combination of residual epicatechins and neo‐formed compounds. This supports our findings that while specific thermolabile molecules may degrade, the overall bioactive profile is reconfigured toward a more potent antioxidant status through the release of bound precursors and the development of Maillard‐derived products.

A review published by Jiang et al. (2023) confirms that (poly)phenolic molecules can undergo structural transformations into smaller phenolic derivatives under high‐temperature conditions. Specifically, while roasting and drying processes have been shown to increase the concentration of trans‐catechins, there is a simultaneous decrease in both galloylated and non‐galloylated catechins. Conversely, the content of gallic acid increases significantly. This phenomenon may be attributed to the isomerization and decarboxylation of catechins, as well as the thermal hydrolysis of complex gallotannins during processing. Such chemical evidence supports the observed increase in gallic acid concentrations in roasted USA, Iranian, and European pistachio samples. Jiang et al. (2021) similarly observed that gallic acid levels are positively correlated with the intensity of the roasting process in tea leaves. Conversely, more complex molecules, such as epicatechins, are inversely affected by thermal treatment, as they undergo significant degradation facilitated by the Strecker degradation reaction. The degradation of these larger polyphenolic structures likely contributes to the liberation of simpler units, such as gallic acid, thereby explaining the increased concentration of the latter in dry roasting‐like treatment pistachio samples.

**FIGURE 3:**
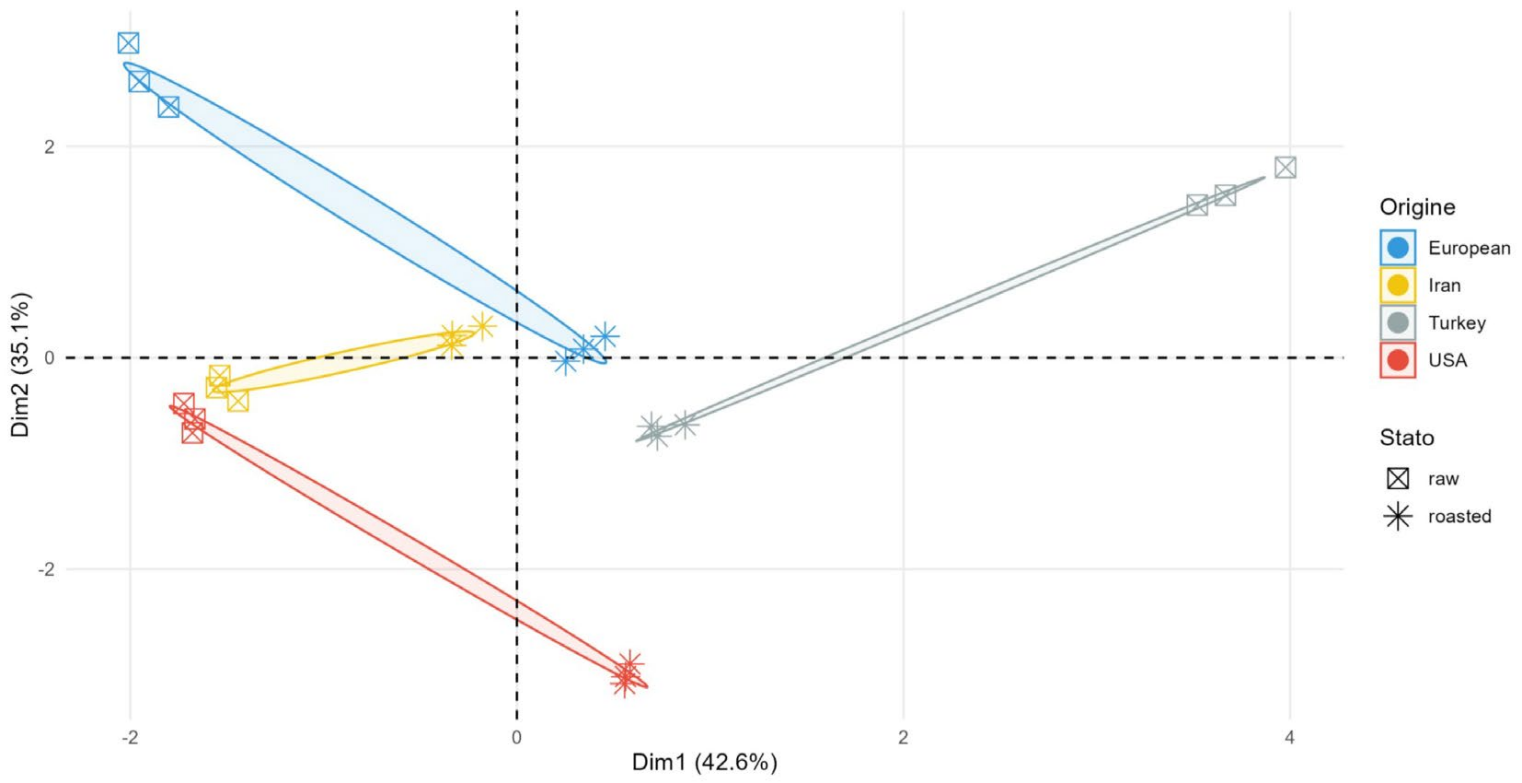
PCA biplot illustrating the discrimination of pistachio samples by origin and thermal treatment based on (poly)phenol differences.

This complex and compound‐dependent behavior underscores that the net effect of roasting on phenolic composition is highly influenced by both geographical origin and cultivar‐specific characteristics. Consequently, processing effects should not be generalized across all pistachio sources, as the interaction between genotype, environmental conditions, and thermal treatment plays a decisive role in determining the final (poly)phenolic profile (Chandrasekara and Shahidi 2011; Locatelli et al. 2015; Pedron et al. 2025; Pelvan et al. 2012; Rodríguez‐Bencomo et al. 2015; Younis et al. 2024). To better evaluate the chemical differentiation in (poly)phenol composition among the geographical origins before and after roasting of the samples, a multivariate statistical approach was employed Figure 3.

The PCA effectively discriminates among pistachio samples based on their geographical origin and processing status, with the first two dimensions accounting for accumulative 77.7% of the total variance. The first dimension (Dim1, 42.6%) primarily illustrates the geochemical signature of the samples, revealing a distinct separation for Turkish pistachios. This segregation is largely attributed to the significantly higher concentrations of gallic acid and increased antioxidant capacity observed in the Turkey group compared to other origins. Conversely, the second dimension (Dim2, 35.1%) characterizes the chemical modifications induced by thermal treatment, where a consistent downward shift is observed for all samples transitioning from a raw to a roasted state. The roasting process promotes the degradation of complex structure, as reported by several studies (Antony and Farid 2022; Jiang et al. 2021; Mantia et al. 2023), facilitating the liberation and detection of specific phenolics, as evidenced by the notable increase in gallic acid within the Iranian roasted samples. Despite these processing‐induced shifts, the 95% confidence ellipses confirm that geographical origin remains the dominant factor in determining the overall (poly)phenol profile, as the clusters for Turkey, Iran, USA, and European samples remain largely non‐overlapping throughout the processing stages. These results suggest that while roasting alters individual nutrient concentrations, it does not obscure the inherent chemical “fingerprint” associated with the geographical provenance of the nuts.

### 3.2 Antioxidant Capacity

The total antioxidant capacity, measured by the ABTS and DPPH assays (Table 3), yielded differing results. For DPPH assay, no significant difference was registered among the Iranian, European, and USA raw samples, while the Turkish samples exhibited the highest antioxidant capacity, reaching 3.73 mg TE/g dw in the DPPH assay. In comparison, the ABTS assay shows a similar antioxidant level for Iranian and USA raw samples; the highest is confirmed for the Turkish raw sample: 1.05 mg TE/g dw.

**TABLE 3:** Antioxidant capacity estimate with ABTS and DPPH assay (mg TE/g dw) and the relative change (Δ% ABTS; Δ% DPPH).

Sample	ABTS		DPPH	
I	0.323 ± 0.003^c^		0.419 ± 0.04^b^	
E	0.305 ± 0.01^b^		0.849 ± 0.04^b^	
U	0.279 ± 0.03^c^		0.285 ± 0.03^b^	
T	1.05 ± 0.08^a^		3.73 ± 0.1^a^	

	ABTS	Δ% ABTS	DPPH	Δ% DPPH
IR	0.352 ± 0.05^b^	+9.2	+0.901 ± 0.08^b^	+114.9
ER	0.535 ± 0.03^b^	+75.3	+1.09 ± 0.02^b^	+27.9
UR	0.408 ± 0.003^b^	+46.2	+0.555 ± 0.01^b^	+95.2
TR	2.15 ± 0.2^a^	+105.6	+13.2 ± 0.4^a^	+254.9

*Note:* Results are reported as the mean ± standard deviation. Statistical significance is indicated by different letters for each column, where values sharing the same letter are not significantly different (*p* < 0.05). Origin codes: I, Iran; E, Spain; U, USA; T, Turkey; IR, dry roasting‐like treatment Iran; ER, dry roasting‐like treatment Spain; UR, dry roasting‐like treatment USA; TR, dry roasting‐like treatment Turkey.

**FIGURE 4:**
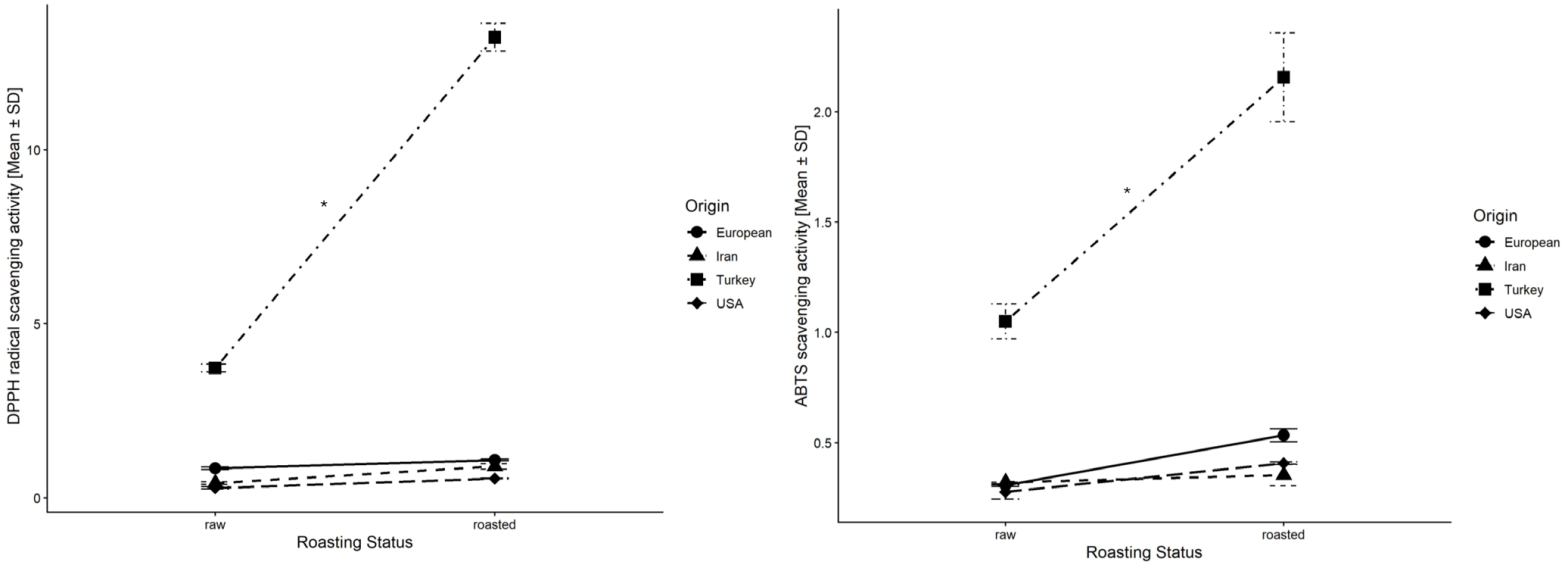
Interaction effect of geographic origin and roasting process on DPPH and ABTS assay (mg TE/g dw). Data are presented as mean standard deviation (*n* = 3). Asterisks indicate significant differences between raw and roasted status within the same geographic origin (*p < 0.01), as determined by Two‐way analysis of variance (ANOVA) followed by Tukey's post hoc test. ABTS left plot, DPPH right plot.

Regarding the antiradical scavenging activity determined by the DPPH assay, the Turkish sample exhibited the most substantial improvement upon roasting, with an increase of +257% compared to its raw state, as reported by the interaction plot above (Figure 4). The Iranian and USA samples also demonstrated a significant increment of +115% and +95%, whereas the European pistachios showed a compatibly modest yet significant increase of 28%. This consistent enhancement of antioxidant capacity across all matrices suggests a general positive effect of thermal processing. Rodríguez‐Bencomo et al. (2015) reported this same trend in pistachios, noting a progressive increase in antioxidant capacity measured in mg TE/g. Specifically, the raw samples exhibited a mean value of 2.01 mg TE/g, which rose to 2.44 mg TE/g after an initial roasting step (160°C for 20 min), and further to 2.88 mg TE/g after a subsequent identical treatment. This progressive data indicates a slight cumulative rise in DPPH radical‐scavenging values with extended thermal treatment. This trend is not limited to pistachios; similar increases in antioxidant activity have been documented in other nuts, such as chestnuts. Mustafa et al. (2021) reported a similar behavior in chestnuts, where roasting elevated antioxidant capacity from 2.81 to 3.37 mg TE/g (Mustafa et al. 2021). This enhancement is often attributed both to the increased Total Radical‐scavenging Capacity (TRC) and to the formation of melanoidin pigments via the Maillard reaction during heating. Conversely, not all nuts respond similarly to roasting; the processing of Brazilian baru nuts, for instance, revealed a contrasting effect, showing a notable decrease in antioxidant capacity from 0.72 to 0.37 mg TE/g.

Similarly, assessment of the antioxidant capacity using the ABTS radical cation decolorization assay revealed that the Turkey sample exhibited the most substantial enhancement following roasting, with an increase of 106% relative to its unprocessed samples. The European pistachios registered the second‐highest increase displaying a 75% rise in ABTS scavenging capacity compared to the raw samples, a result that contrasts notably with their comparatively modest increase in DPPH assay. Following these were the USA sample, with an increase of 46%, and finally, the Iranian pistachios, which showed the most modest ABTS improvement at +9%. The current results, which indicate an enhanced antioxidant capacity after roasting, are consistent with the literature, particularly the findings of Hojjati et al. (2015). They documented a strong positive effect of hot‐air roasting on the antioxidant capacity of pistachios, citing a significant rise from 1.67 mg TE/g in the raw sample to 2.96 mg TE/g post‐roasting. This 77% increase in TE highlights the considerable impact that thermal processing can have on releasing and/or generating antioxidant compounds within the food matrix.

The thermal enhancement of antioxidant capacity is primarily explained by the generation of new reducing compounds and the release of bounded phenolics compounds. In fact, roasting initiates the Maillard reaction and the formation of Maillard reaction products. These compounds, including pyrroles, melanonids, and furans, often possess structures with high electron‐donating capacity, mimicking phenolic compounds and contributing directly to the observed increase in TRC (Pedron et al. 2025; Yanagimoto et al. 2002). Also, the high temperatures exert a destructive effect on the food matrix. This thermal stress causes the degradation of complex molecules, such as tannins, into smaller, more active phenolic units. Crucially, heating also breaks the bonds between phenolic compounds and macromolecules such as proteins and lignocellulosic structures. This release effectively increases the extractability of total phenolic compounds and increases the antioxidant capacity, thereby boosting the measured antiradical capacity (Boateng et al. 2008; Garrido et al. 2008; Hojjati et al. 2015; Lemos et al. 2012; Mustafa et al. 2021; Rodríguez‐Bencomo et al. 2015).

It is worth noting that while the thermal conditions (140°C) enhanced the antioxidant capacity through the synthesis of new molecules, these same Maillard reaction pathways can lead to the generation of process‐induced contaminants, such as acrylamide. Although the quantification of such compounds was beyond the current analytical scope, our findings highlight the importance of balancing bioactive liberation with chemical safety in pistachio processing.

The radical scavenging capacity observed in raw pistachio samples, as measured by ABTS and DPPH assays, can be directly correlated with the individual phenolic compounds quantified via HPLC‐DAD (Table 2) and the TPC (Table 1). In the raw matrix, molecules such as gallic acid, protocatechuic acid, and p‐hydroxybenzoic acid likely serve as the primary drivers of the antioxidant response.

In contrast, the significant increase in antioxidant capacity following the roasting process suggests a shift in the bioactive profile. While some native (poly)phenols undergo thermal degradation, the net increase in antioxidant activity may be attributed to the synthesis of new neo‐formed compounds during the Maillard reaction. These high‐molecular‐weight products often exhibit potent radical scavenging abilities, effectively compensating for the loss of thermolabile phenolic structures. This dual mechanism explains why the roasted samples maintain or exceed the antioxidant power of their raw counterparts, despite the reduction in specific individual polyphenols.

### 3.3 Total Content of Chlorophyll and Carotenoids

The color characteristics of pistachios, primarily governed by the presence of chlorophylls (a and b) and carotenoids, constitute a key quality parameter influencing consumer appeal and market acceptance. Beyond visual quality, these pigments are significant due to their strong antioxidant capacity and their established role as beneficial dietary compounds linked to the prevention of cardiovascular disease. Therefore, color analysis is essential for assessing both the visual marketability and the inherent nutritional and functional integrity of the final product (Bellomo and Fallico 2007; Pumilia et al. 2014; Stahl and Sies 2003).

**FIGURE 5:**
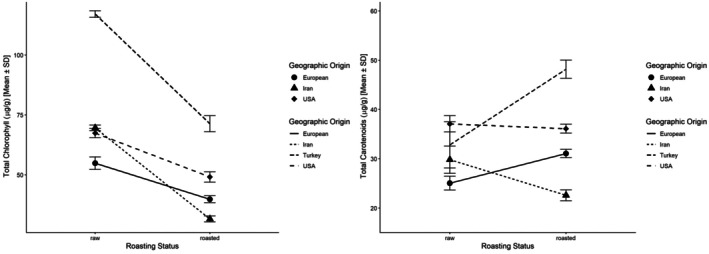
Interaction plot showing the effect of roasting status (raw vs. roasted) on total chlorophyll content (left) and carotenoids (right) (mg/kg dw) across samples from different geographic origins. Symbols represent the mean value, and error bars represent the standard deviation (*n* = 3). Asterisks indicate significant differences between raw and roasted status within the same geographic origin (*p < 0.01), as determined by Two‐way analysis of variance (ANOVA) followed by Tukey's post hoc test.

The results of total chlorophyll and total carotenoids concentrations in raw and dry roasting‐like treatment pistachios (Table 4), determined by spectrophotometric assay, align with the findings reported by Dini et al. (2019). Moreover, Figure 5 displays the interaction plots for total chlorophyll and carotenoid content.

**TABLE 4:** Total content of chlorophyll a and b and carotenoid (mg/kg dw) and the relative change (Δ% chlorophyll a; Δ% chlorophyll b; Δ% chlorophyll a/b; Δ% Σ chlorophyll a + b; Δ% carotenoids).

Sample	Chlorophyll a		Chlorophyll b		Chlorophyll a/b		Σ Chlorophyll a + b		Carotenoids	
I	40.1 ± 2^b^		39.4 ± 2^b^		1.37 ± 0.2^b^		79.9 ± 1^b^		29.8 ± 3^ab^	
E	30.9 ± 0.7^c^		23.9 ± 0.8^b^		1.29 ± 0.1^b^		54.8 ± 3^c^		25.05 ± 2^b^	
U	50.4 ± 0.4^c^		35.4 ± 2^a^		0.903 ± 0.1^c^		85.9 ± 2^b^		37.1 ± 1^a^	
T	76.4 ± 3^a^		40.6 ± 2^a^		1.89 ± 0.2^a^		117 ± 1^a^		32.8 ± 1^a^	

	Chlorophyll a	Δ% Chlorophyll a	Chlorophyll b	Δ% Chlorophyll b	Chlorophyll a/b	Δ% Chlorophyll a/b	Σ Chlorophyll a + b	Δ% Σ Chlorophyll a + b	Carotenoids	Δ% Carotenoids
IR	20.7 ± 0.9^c^	−48.31	10.8 ± 2^c^	−63.09	1.94 ± 0.3^a^	41.26	31.6 ± 1^d^	−54.57	22.6 ± 1^d^	−24.20
ER	22.2 ± 3^c^	−28.10	17.6 ± 3^b^	−26.48	1.26 ± 0.1^b^	−2.27	39.8 ± 2^c^	−27.39	31.1 ± 5^c^	+24.02
UR	31.9 ± 0.8^b^	−36.61	17.2 ± 0.9^b^	−51.63	1.88 ± 0.3^a^	+108.39	49.1 ± 2^b^	−42.81	36.1 ± 1^b^	−2.63
TR	42.3 ± 2^a^	−44.68	29.1 ± 2^ba^	−28.51	1.45 ± 0.02^ab^	−23.06	71.3 ± 3^a^	−39.06	48.2 ± 2^a^	+46.79

*Note:* Data are presented as mean ± standard deviation (*n* = 3). Statistical significance is indicated by different letters (a, b, c, etc.) for each column, where values sharing the same letter are not significantly different (*p* < 0.05). Origin codes: I, Iran; E, Spain; U, USA; T, Turkey; IR, dry roasting‐like treatment Iran; ER, dry roasting‐like treatment Spain; U, dry roasting‐like treatment USA; T, dry roasting‐like treatment Turkey.

These results collectively affirm that geographical origin significantly influences both the profile and concentration of the analyzed pigments. Specifically, the data revealed that pistachios from Turkey exhibited the highest concentration of total chlorophyll (a + b) at 117 mg/kg, followed by samples from the USA, Iran, and Europe. Conversely, the highest concentration of total carotenoids (27 mg/kg) was recorded in the USA samples, followed by the Turkish, Iranian, and European counterparts.

In literature, lutein is consistently identified as the predominant carotenoid in pistachios, with reported concentrations ranging from 18 to 52 mg/kg. This substantial variability is likely attributable to differences in cultivars, geographical provenance, and post‐harvest processing methods (Bellomo and Fallico 2007; Pumilia et al. 2014; Rabadán et al. 2018). The accumulation of pigments and chlorophylls in the seed is modulated by multiple factors; for instance, Gebregziabher et al. (2023). demonstrated that carotenoid content in soybeans is determined by genotype (accounting for 78% of the variation), as well as environmental and climatic conditions. Similarly, Zhao et al. (2026) reported that color and carotenoid profiles in foxtail millet are strongly genotype‐dependent, with concentrations varying between 5.4 and 19 mg/kg across different cultivars.

Beyond genetic factors, pedoclimatic variables (such as seasonal temperature regimes, precipitation levels, and solar radiation intensity) collectively influence pigment abundance (chlorophylls and carotenoids) by regulating physiological pathways and gene expression. do Nascimento‐Silva et al. (2026) confirmed that carotenoid content and chemical fingerprints in *C. brasiliense* pulp are closely linked to geographical area, soil characteristics, and specific agronomic practices. Taken together, these findings demonstrate that the European samples consistently possessed the lowest pigment concentrations when compared to pistachios harvested in the USA or Turkey.

The chlorophyll a/chlorophyll b ratio exhibited a consistent predominance of chlorophyll a across all analyzed samples. This observation aligns with previously reported trends, particularly those of Boukid et al. (2019), who reported similar ratios, ranging from 0.80 to 1.5 in thirty‐six pistachio samples sourced from diverse geographical locations, including Italy, California, Iran, Syria, Kyrgyzstan, and Turkey. According to these authors, a higher chlorophyll a/b ratio is generally associated with superior pistachio quality, as it reflects a great abundance of photosynthetically active pigments and potentially enhanced oxidative stability during storage and processing. The observed variations in pigment concentration are supported by various preceding studies. For instance, Bellomo and Fallico (2007) and Boukid et al. (2019) have previously established that factors such as geographical origin, cultivar, harvest and post‐harvest conditions, and the grade of ripeness are critical determinants of the final concentrations of both total chlorophyll and total carotenoids in pistachios. The current data, therefore, reinforce the established understanding of the multi‐factorial influences on pistachio quality metrics.

Roasting resulted in a drastic decrease in the concentration of both chlorophyll a and chlorophyll b across all pistachio samples. The total chlorophyll degradation was most pronounced in the Iranian sample (−55%), followed by the USA (−43%), Turkey (−39%), and European (−27%) samples. This differential degradation suggests that initial chlorophyll concentrations, matrix effects, or specific roasting protocols may influence heat stability. Examining the individual chlorophyll components, the Iranian sample exhibited the greatest loss for both chlorophyll a (−48%) and chlorophyll b (−63%). Substantial losses were also observed in the USA sample (chlorophyll a: −37%; chlorophyll b: −52%) and the Turkey sample (chlorophyll a: −45%; chlorophyll b: −29%). The European sample demonstrated the lowest degradation for both components (chlorophyll a: −28%; chlorophyll b: −27%).

The chlorophyll a/b ratio remained largely stable after roasting in the European and USA pistachio samples. However, the ratio displayed contrasting responses in the other two origins. Specifically, the dry roasting‐like treatment Iranian pistachios registered an increase in the a/b ratio, indicative of a greater relative degradation of chlorophyll b compared to chlorophyll a. In contrast, the Turkey sample exhibited a decrease in this ratio, suggesting that chlorophyll a was preferentially thermally degraded. This overall decline in chlorophyll concentration is consistent with established literature, where heat treatment is known to promote the degradation of chlorophyll a and b via the displacement of the central Mg^2+^ ion, forming various derivatives such as pheophytin a and b, and pyro‐pheophytin *a* and *b* (Pumilia et al. 2014). Similar thermal degradation has been widely reported in other foodstuffs, including fruits and vegetables (Dini et al. 2019; Heaton and Marangoni 1996).

Interesting data were obtained from the quantification of carotenoids after the roasting process. The Iranian sample registered a decrease in total carotenoid concentration, falling from 29.8 to 22.6 mg/kg. Conversely, the USA sample showed non‐significant changes in its carotenoid content. Most notably, a substantial increase in carotenoid concentration was observed in the European (+24%) and Turkey (+47%) samples. The increasing trend in carotenoids, following heat treatment, is supported by previous studies, which confirm the relative resistance of these pigments to thermal degradation. Furthermore, the application of heat can lead to the inactivation of endogenous enzymes capable of oxidizing carotenoids, which may contribute to the observed net increase or preservation of their concentrations (Henry et al. 1998; Rabadán et al. 2018; Shang et al. 2025).

### 3.4 Multivariate Analysis

**FIGURE 6:**
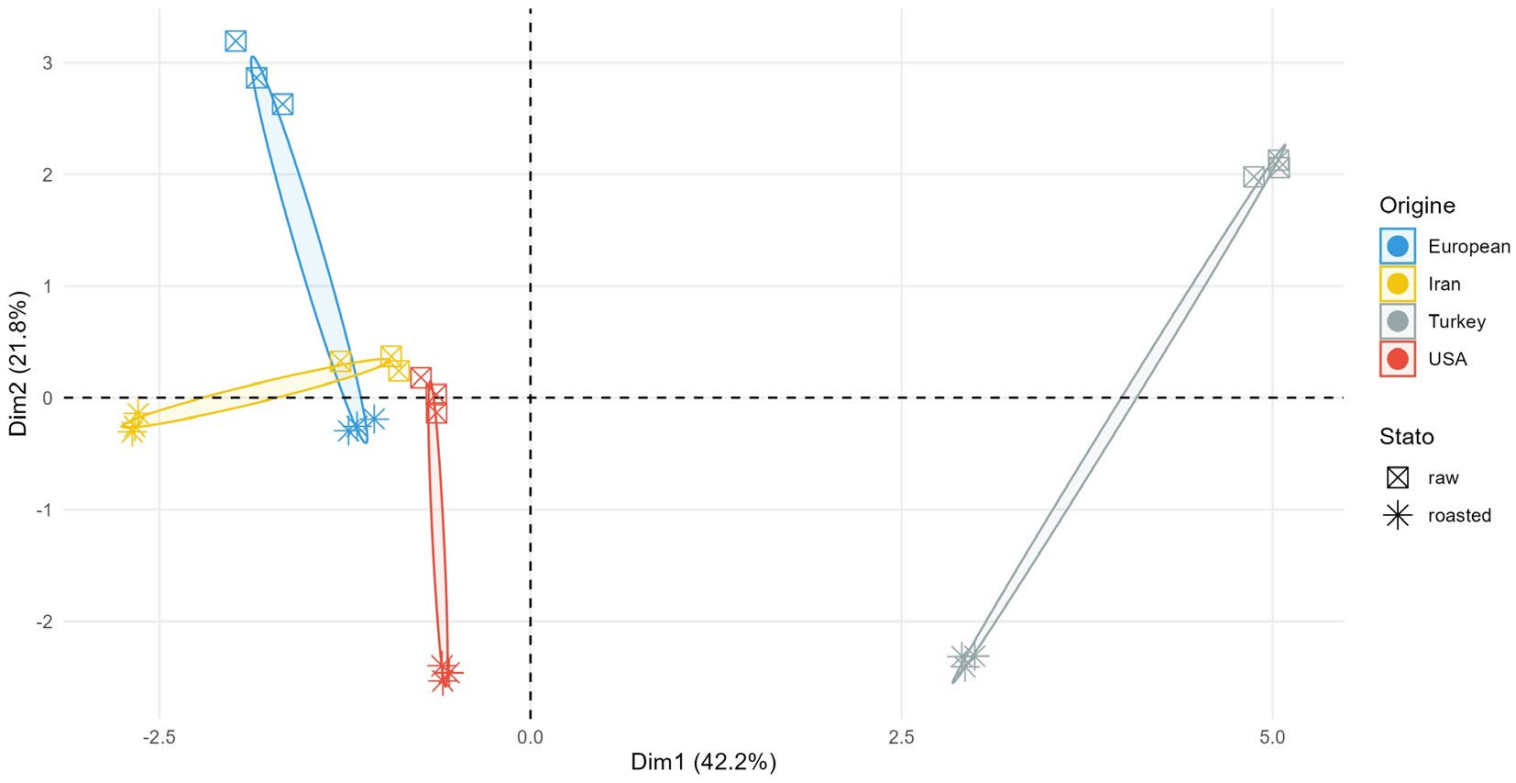
PCA biplot illustrating the discrimination of pistachio samples by origin and thermal treatment based on biochemical profile.

To evaluate the interrelationships among the phytochemical profiles and antioxidant activities (DPPH, TPC, ABTS, individual polyphenols, chlorophylls, and carotenoids) across different pistachio origins and processing stages, a Principal Component Analysis was performed (Figure 6). The multivariate analysis was executed using R software (v. 4.4.2), specifically leveraging the FactoMineR and factoextra packages for computation and graphical rendering. Data were standardized (unit variance scaling) prior to analysis to ensure equal weighting of variables with different magnitudes. Confidence ellipses (95%) were calculated based on the geographical origin to assess group separation, while processing status (raw vs. dry roasting‐like treatment) was highlighted using distinct point shapes.

The multivariate analysis revealed that raw samples from the USA and Iran exhibited a high degree of similarity, clustering closely together. In contrast, European and Turkish raw samples appeared as distinct clusters, suggesting that raw pistachios possess a unique chemical fingerprint dictated by their geographical provenance.

The observed variations in (poly)phenol composition, antioxidant capacity, and pigment concentration appear strongly dependent on geographical origin as reported in PCA (Figure 6). According to Mainente et al. (2023), several independent variables within the production area such as pedoclimatic conditions, soil mineral composition, and the maturation stage at harvest, significantly influence these nutraceutical properties. These environmental factors act as external stressors that can trigger the phenylpropanoid pathway, leading to the differential accumulation of protective phenolic compounds. This geographical fingerprint is consistent with findings by Mir‐Cerdà et al. (2025) who demonstrated that the metabolomic profile of plant matrices (e.g., olive leaves) often clusters more closely by geographical provenance than by cultivar. This suggests that the local terroir and pedoclimatic characteristics exert a predominant influence on the phenolic phenotypic expression, potentially overshadowing genetic factors in certain environments. Kurt and Karakaya (2025) attributed variations in the antioxidant capacity of hazelnuts harvested across different Turkish regions to differences in light exposure, UV radiation intensity, and ambient temperature, among other environmental stressors. Furthermore, agronomic variables, including specific cultivation practices, irrigation regimes, and cultivars, have been shown to significantly modulate the synthesis of bioactive secondary metabolites. These findings suggest that the antioxidant potential of pistachios is the result of a complex interaction between the genotype and the environment. Therefore, subsequent longitudinal studies are warranted to further elucidate the synergistic links between specific varieties, agronomical interventions, and the resulting antioxidant profiles of pistachio cultivars.

The roasting process induced a significant shift in the metabolic profile for all origins. Notably, all dry roasting‐like treatment samples migrated toward the lower quadrant of the score plot, indicating a profound reconfiguration of their bioactive composition. Interestingly, the dry roasting‐like treatment European samples shifted to a position chemically similar to the raw Iranian and USA samples. Despite these drastic changes, each geographical group maintained its own cluster integrity after roasting. This demonstrates that while thermal treatment fundamentally alters the pistachio matrix, the underlying influence of geographical origin remains detectable, allowing for continued sample differentiation.

## 4 Conclusions

This study demonstrates that geographical origin is the primary determinant of the baseline phytochemical profile in pistachios, significantly influencing the trajectory of compositional changes during thermal processing at 140°C. While roasting induces the degradation of thermolabile chlorophylls and decreases (poly)phenol concentrations in high‐content samples. Notably in Turkish pistachios (from 665 to 241 μg/g), it results in a net increase in total antioxidant capacity across all origins, with the most pronounced enhancement observed in Turkish samples (+255% TPC and DPPH). These shifts are characterized by the systemic increase of gallic acid and the preservation of carotenoids, alongside the formation of Maillard‐derived products. Ultimately, roasting reconfigures the pistachio's bioactive landscape into a more potent antioxidant matrix regardless of initial origin. These findings provide a scientific basis for promoting dry roasting‐like treatment pistachios as a functional food and offer a comparative framework for the food industry to optimize the nutritional value of kernels from the four major global production regions.

### 4.1 Future Perspectives

Although this study establishes the chemical and antioxidant foundation of dry roasting‐like treatment pistachios, further research is required to correlate these compositional shifts with consumer perception. Future investigations should incorporate sensory panel evaluations to assess how the reconfiguration of (poly)phenols and the formation of Maillard‐derived volatiles and non‐volatile compounds impact the flavor, bitterness, and overall palatability of the nuts. Such studies will be essential to bridge the gap between chemical potency and consumer acceptance in the functional food market.

While this study characterizes the immediate enhancement of the antioxidant profile following roasting, it provides the necessary baseline for future investigations into the oxidative stability of these samples. Further research should focus on how the thermal reconfiguration of (poly)phenols and the degradation of pigments influence the long‐term quality and lipid protection of pistachios during commercial storage.

Finally, a limitation of this study is the lack of certified mono‐cultivar documentation for each sample; therefore, while geographical trends were identified, future research utilizing genetically verified varieties is required to definitively isolate the influence of cultivar from environmental and processing factors.

## Author Contributions

**G. Barbieri:** investigation, writing – original draft, formal analysis. **R. Perestrelo:** formal analysis, methodology, writing – review and editing, data curation. **J. S. Câmara:** conceptualization, project administration, supervision, validation, visualization, writing – review and editing. **Y. Jaouhari:** writing – original draft, writing – review and editing. **M. Bordiga:** conceptualization, project administration, supervision, validation, writing – review and editing. **G. Pedron:** data curation, formal analysis, investigation, methodology, writing – original draft, writing – review and editing.

## Conflicts of Interest

The authors declare no conflicts of interest.

## Data Availability

The data that support the findings of this study are available from the corresponding author upon reasonable request.
